# Endoscopic Surgery for Facial Lipoma Excision

**DOI:** 10.1155/DTE.7.181

**Published:** 2001

**Authors:** H. Koike, H. Kitano, M. Fujimura, T. Kinoshita, H. Kataoka, M. Hirano, S. Seno, K. Kitajima

**Affiliations:** 1 Second Department of Surgery Shiga University of Medical Science Otsu Shiga 520-229-2 Japan; 2 Department of Otolaryngology and Head and Neck Surgery Shiga University of Medical Science Otsu Shiga 520-229-2 Japan; 3 Department of Otolaryngology Head and Neck Surgery Faculty of Medicine Tottori University, 36-1 Nishimachi Yongao 683-8504 Japan

## Abstract

Minimally invasive endoscopic surgery in the neck, first reported by Gagner in 1996, has been adopted by a number of other surgical specialties. We have developed new techniques for performing endoscopic enucleation lipoma. Using our new techniques, various complications, such as injury to nerves and vessels, are prevented. The technique generates cosmetically satisfying results. Expansion of minimally invasive surgery into the facial area will be enhanced by the future development of instruments for this area, and decrease operating time and hospital stay.


*Diagnostic and Therapeutic Endoscopy*, Vol. 7, pp. 181–185
Reprints available directly from the publisher
Photocopying permitted by license only

© 2001 OPA (Overseas Publishers Association) N.V.
Published by license under
the Harwood Academic Publishers imprint,
part of Gordon and Breach Publishing
a member of the Taylor & Francis Group.
All rights reserved.

### Endoscopic Surgery for Facial Lipoma Excision

H. KOIKE[a], H. KITANO[b]*, M. FUJIMURA[a], T. KINOSHITA[a], H. KATAOKA[b], M. HIRANO[a], S. SENO[b] and K. KITAJIMA[b]

[a]*Second Department of Surgery;* [b]*Department of Otolaryngology and Head and Neck Surgery, Shiga University of Medical Science, Otsu, Shiga 520-229-2, Japan*

*(Received 27 March 2001; Revised 14 May 2001; In final form 14 May 2001)*

Minimally invasive endoscopic surgery in the neck, first reported by Gagner in 1996, has been adopted by a number of other surgical specialties. We have developed new techniques for performing endoscopic enucleation lipoma. Using our new techniques, various complications, such as injury to nerves and vessels, are prevented. The technique generates cosmetically satisfying results. Expansion of minimally invasive surgery into the facial area will be enhanced by the future development of instruments for this area, and decrease operating time and hospital stay.

*Keywords*: Endoscopic surgery; Facial lipoma; Mandibular lipoma; Minimally invasive surgery

Minimally invasive endoscopic surgery in the neck, first reported by Gagner in 1996, has been adopted by a number of other surgical specialties [1]. We began performing minimally invasive endoscopic surgery for the treatment of benign tumors in the neck in 1998 [2]. In this paper, we report our first endoscopic facial lipoma excision.

### CASE REPORT

The patient was a 38-year-old woman referred to our hospital for a lump at the right side of the mandible in 1995. The lump was soft and movable, and 2 cm in diameter. We recommended surgical therapy, but the patient declined in order to avoid the resultant facial scars, subsequently, the lump enlarged. She visited our hospital in April 1999 desiring surgical therapy. The lump was 6 cm × 5 cm, soft and movable (Fig. 1). A CT scan demonstrated a low density round mass at the right mandible. An MRI scan demonstrated a mass of high intensity on T1 weighted images and low intensity on T2 weighted images (Fig. 2). Fine needle aspiration biopsy revealed class I cytology with lipid-laden cells. These examinations suggested that the tumor was a lipoma.

*Corresponding author. Current address: Department of Otolaryngology, Head and Neck Surgery, Faculty of Medicine, Tottori University, 36-1 Nishimachi, Yongao, Japan 683-8504. Tel.: +81-859-34-8123. Fax: +81-859-34-8090. E-mail: hkitano@grape.med.tottori-u.ac.jp

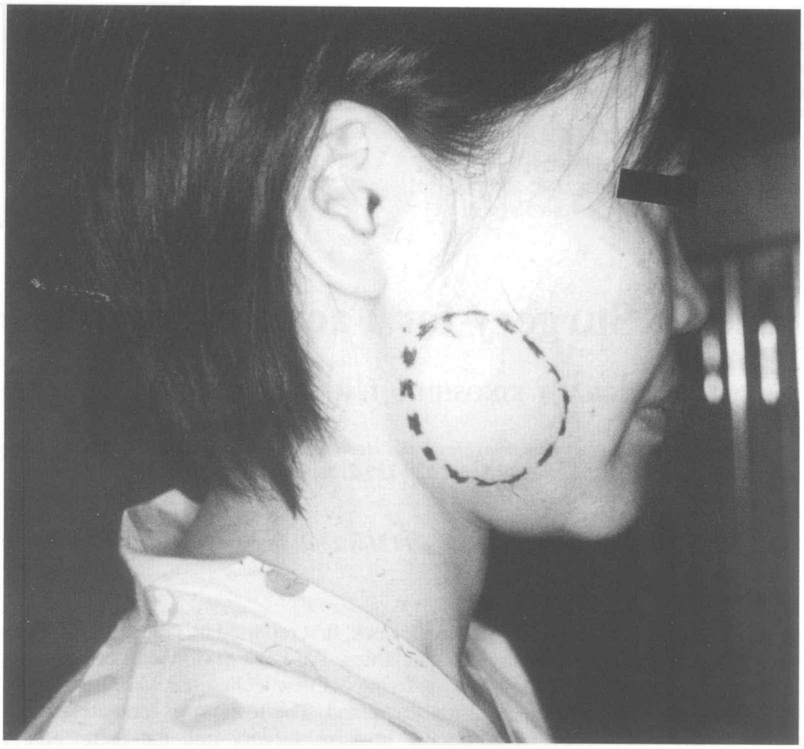

FIGURE 1 Preoperative photograph of a patient with facial lipoma, demonstrating a right mandibular mass. The mass was 6 cm long and 5 cm wide.

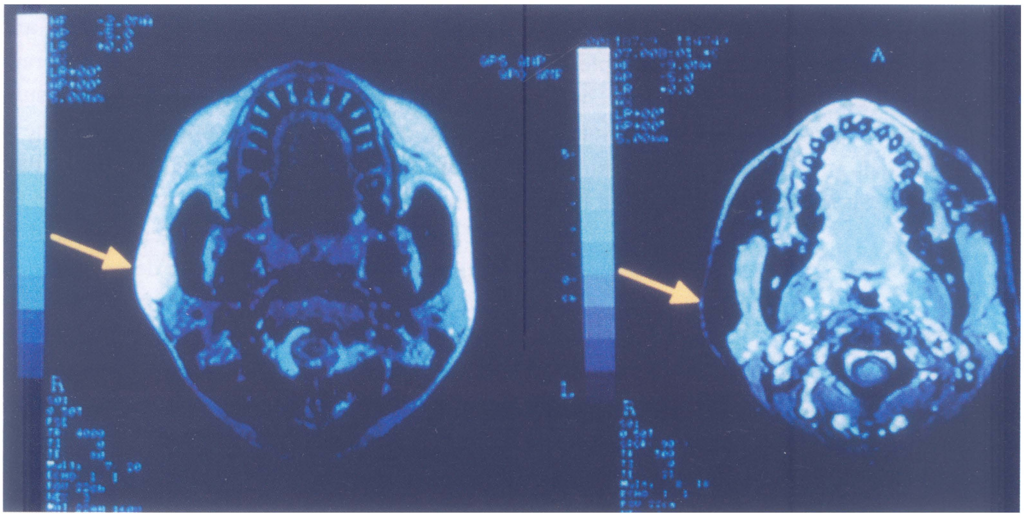

FIGURE 2 The arrow indicates the mass. An MRI scan demonstrated a high intensity mass on T1 weighted images (right), and a low intensity mass on T2 weighted images (left). These results suggested the mass was lipoma.

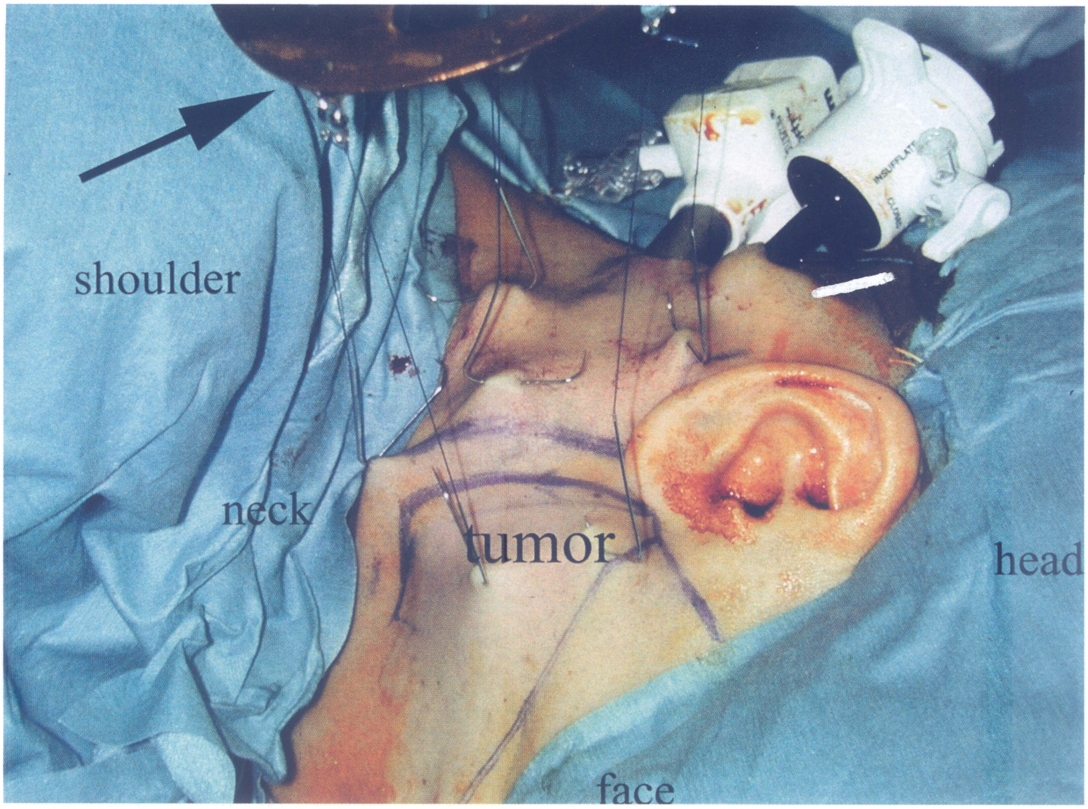

FIGURE 3 Hooks lifted the neck skin over the lump (black arrow). A 10 mm endoscope was inserted through the 10 mm trocar. A 5 mm incision was made in the right upper neck. Through the 5 mm incision, a 5 mm trocar was inserted for washing and suct[illegible]ning.

Surgical options were discussed with the patient according to standard practices of the Ethics Committee of Shiga University of Medical Science. This included discussion of possible complications resulting from endoscopic neck surgery, including subcutaneous emphysema and facial palsy. Informed consent was obtained. The patient chose to undergo endoscopic rather than conventional open surgery.

### SURGICAL TECHNIQUE

The patient was placed in a left lateral recumbent position. The operation was performed under general anesthesia. Two 1 cm skin incisions were placed in the hair-bearing area at the post-inferior auricular region, so as to be covered by her hair. Through these incisions, a 10 mm endoscope and surgical instruments were inserted. A 5 mm incision was made in the right upper neck. Through the 5 mm incision, a 5 mm trocar was inserted for washing and suctioning. Blunt dissection was carried out under the skin toward the mass. We created an open space by blunt dissection and lifted the skin using hooks to avoid the complications of carbon dioxide insufflation (Fig. 3) [3]. Endoscopic approach advised for superficial preparotid neoplasm. The tumor was abutting on the parotid gland, and was densely adherent to the surrounding tissue. The tumor was resected by bipolar scissors. Injury to the right marginalis mandibulae nerve was the predominant concern during the operative procedure. Movement of the labii inferioris was confirmed using a facial nerve stimulator (Fig. 4). The main incision (Fig. 1) was extended to 30 mm to remove the tumor. The tumor was confirmed to be a

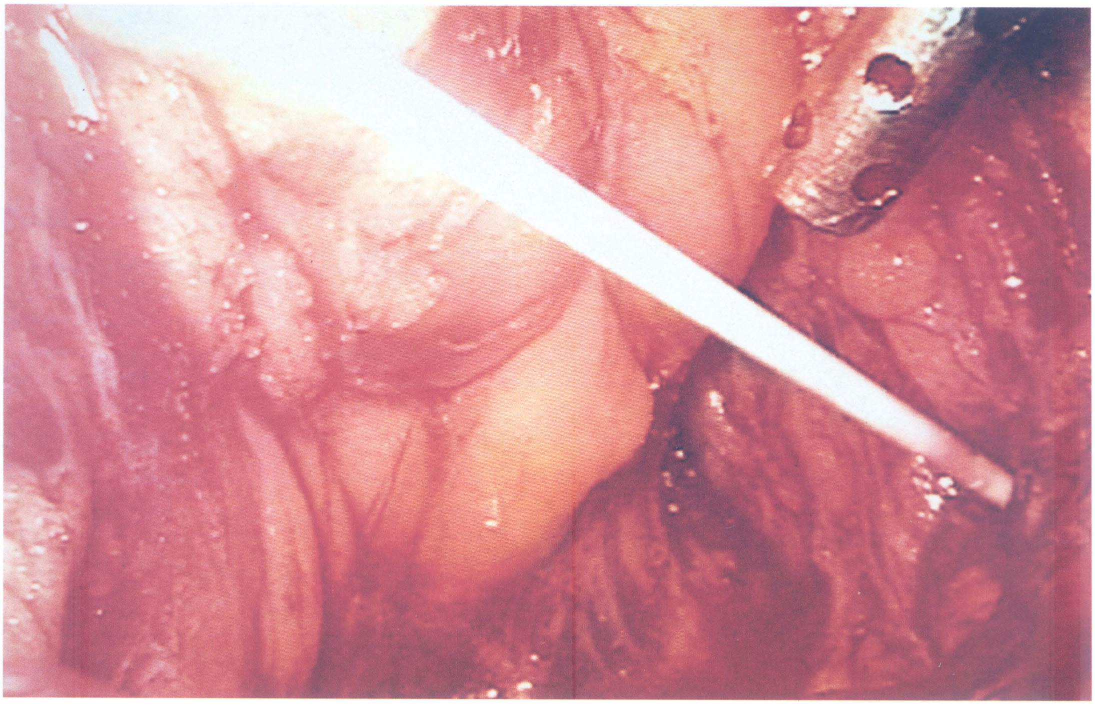

FIGURE 4 The white stick indicates the facial nerve stimulator.

benign lipoma by pathologic examination. A suction drain was left in place and removed the following day. Blood loss was about 40 $cm^3$. The surgical procedure took about 3 h.

### DISCUSSION

Several applications for endoscopic techniques in head and neck surgery have been reported in the past few years. However, as far as we know, there are few reports describing endoscopically assisted excision of benign tumors in the face. This is the first reported case of, not assisted, endoscopic surgery for facial lipoma excision. The surgical technique that we report did not leave visible facial scars, and therefore is ideal for plastic surgery. There are several surgical procedures to excise benign facial tumors. Conventional open surgery leaves big scars, contractures, and keloids on the face. Hallock *et al.* have reported endoscopically assisted suction extraction of lipomas [4,5]. This procedure was cosmetic only, however, due to the inability to confirm complete aspiration of the tumor and its capsule [6].

In developing our new technique, we had several concerns. First, there was a risk of facial nerve paralysis. To avoid facial nerve injury, we used a facial nerve stimulator to confirm movement of the labii inferioris. Second, we needed to create enough space to completely visualize the tumor and capsule. We used hooks to lift the skin of the neck. Using this procedure, we were able to operate. Third, the patient wished to avoid facial scarring, so we made the incisions in the hair-bearing region of the scalp (Fig. 5).

In summary, we have developed a new technique for performing endoscopic enucleation of lipomas. Using our technique, likelihood of complications, such as injury to nerves and vessels, is much lower, and the cosmetic result is superior to conventional procedures. Application of minimally invasive surgery to the facial area will be enhanced by the future development of instruments for this area, and decrease of operating time and hospital stay.

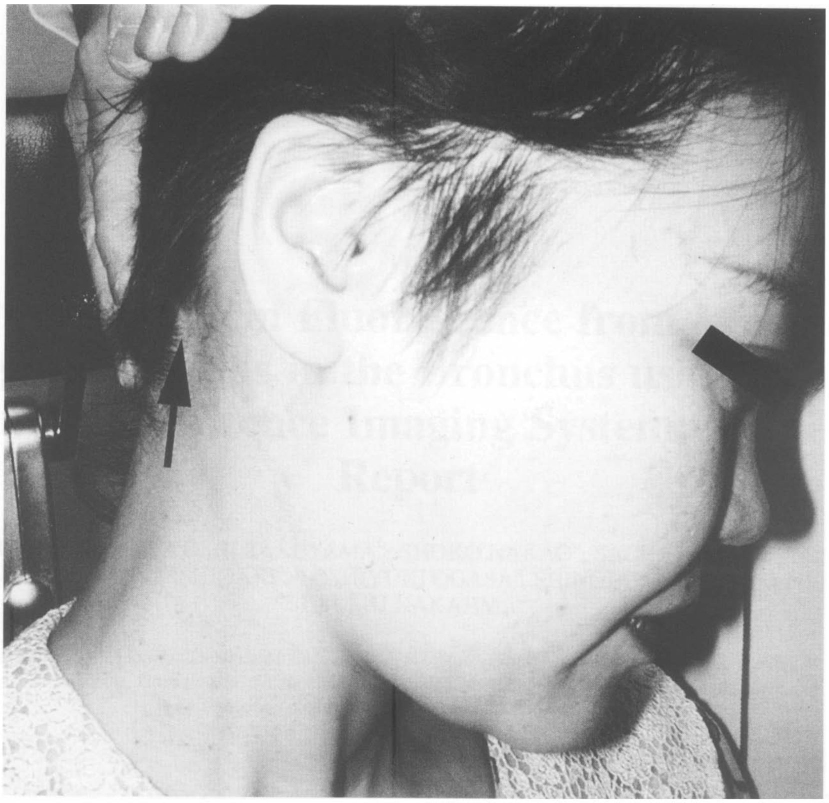

FIGURE 5 Postoperative photograph at 12 weeks. Black arrow indicates scar of incisions.

### *References*

[1] Gagner, M. (1996) "Endoscopic subtotal parathyroidectomy in patients with primary hyperparathyroidism", *Br. J. Surg.* **83**, 875.

[2] Kitano, H., Fujimura, M., Hirano, M., *et al.* (2001) "Endoscopic surgery for lateral cervical cysts", *Surg. Endosc.* **14**, 1086.

[3] Kitano, H., Fujimura, M., Hirano, M., *et al.*, (2000) "Endoscopic surgery for parathyroid functioning adenoma resection—using neck lifting method", *Otolaryngol. Head Neck Surg.* **123**(4), 465–466.

[4] Hallock, G.C. (1995) "Endoscope-assisted suction extraction of lipomas", *Ann. Plast. Surg.* **34**, 32–34.

[5] Hallock, G.C. (1987) "Suction of lipomas", *Ann. Plast. Surg.* **18**, 517.

[6] Nichter, L.S. and Gupta, B. (1990) "Liposuction of giant lipomas", *Ann. Plast. Surg.* **24**, 362.

